# Spectrum of movement disorders in GNAO1 encephalopathy: in-depth phenotyping and case-by-case analysis

**DOI:** 10.1186/s13023-020-01594-3

**Published:** 2020-12-09

**Authors:** Soo Yeon Kim, YoungKyu Shim, Young Joon Ko, Soojin Park, Se Song Jang, Byung Chan Lim, Ki Joong Kim, Jong-Hee Chae

**Affiliations:** 1 Division of Pediatric Neurology, Department of Pediatrics, Pediatric Clinical Neuroscience Center, Seoul National University Children’s Hospital, Seoul, Korea; 2 Rare Disease Center, Seoul National University Hospital, Seoul, Korea; 3 Department of Medicine, Seoul National University College of Medicine Graduate School, Seoul, Korea; 4 Department of Pediatrics, Seoul National University College of Medicine, Seoul, Korea

**Keywords:** *GNAO1*, GNAO1 encephalopathy, Movement disorder, Early-onset dystonia, Early-onset chorea

## Abstract

**Background:**

GNAO1 encephalopathy is a rare neurodevelopmental disorder characterized by distinct movement presentations and early onset epileptic encephalopathy. Here, we report the in-depth phenotyping of genetically confirmed patients with GNAO1 encephalopathy, focusing on movement presentations.

**Results:**

Six patients who participated in Korean Undiagnosed Disease Program were diagnosed to have pathogenic or likely pathogenic variants in *GNAO1* using whole exome sequencing. All medical records and personal video clips were analyzed with a literature review. Three of the 6 patients were male. Median follow-up duration was 41 months (range 7–78 months) and age at last examination was 7.4 years (range 3.3–16.9 years). Initial complaints were hypotonia or developmental delay in 5 and right-hand clumsiness in 1 patient, which were noticed at median age of 3 months (range 0–75 months). All patients showed global developmental delay and 4 had severely retarded development. Five patients (5/6, 83.3%) had many different movement symptoms with various onset and progression. The symptoms included stereotyped hands movement, non-epileptic myoclonus, dyskinesia, dystonia and choreoathetosis. Whole exome sequencing identified 6 different variants in *GNAO1*. Three were novel de novo variants and atypical presentation was noted in a patient. One variant turned out to be inherited from patient’s mother who had mosaic variant. Distinct and characteristics movement phenotypes in patients with variant p.Glu246Lys and p.Arg209His were elucidated by in-depth phenotyping and literature review.

**Conclusions:**

We reported 6 patients with GNAO1 encephalopathy showing an extremely diverse clinical spectrum on video. Some characteristic movement features identified by careful inspection may also provide important diagnostic insight and practice guidelines.

## Introduction

Since the first identification of *GNAO1* as a new causative gene of early-onset epileptic encephalopathy in 2013, variable phenotypes have been reported [1–3]. Epilepsy itself varied from early onset epileptic encephalopathy including Ohtahara syndrome, and generalized and focal epilepsies of different ages [1, 4, 5]. Movement phenotypes were also reported since the first clinical report and have recently become major presenting symptoms: so far, chorea, dystonia, orofacial dyskinesia, and stereotyped hand movements have been reported in patients of different ages, associated with *GNAO1* [1, 6–8]. Developmental milestones are also delayed in most patients with *GNAO1* variants and the degree of developmental delay varies from neonatal hypotonia to intellectual disability [7–9]. Interestingly, most patients mainly present phenotypes between the epilepsy or movement disorder, whereas a small number of patients showed both epilepsy and movement phenotypes equally [10]. Many studies suggested that different locations or different functional changes of variants lead to separate phenotypes [1, 10–12]. Gain-of-function mutation (GOM) turned out to be associated with movement disorder [11]. However, it could not explain all the cases. Further studies on phenotype, genotype, and molecular pathways are certainly required for better understanding. Phenotypes often mimic other neurodevelopmental disorders and are barely immediately recognizable because the disease usually occurs in infancy with nonspecific symptoms and evolves over time. Therefore, comprehensive and serial phenotyping would be the very first step to grasp the disease and establish further functional studies.

Here, we report 6 pediatric patients who carried *GNAO1* variants identified from the Korean Undiagnosed Disease Program (KUDP), with the aim of delineating detailed phenotypes and characterizing their phenotype–genotype association with a comprehensive review of previously reported cases.

## Patients and methods

### Patients and study approval

Six patients who carried *GNAO1* variants were enrolled in this study. All patients were diagnosed using whole exome sequencing (WES) after participating in the KUDP, which launched in 2017 [13]. The entire KUDP protocol including diagnostic process and data sharing was approved by the Institutional Review Board (IRB) of Seoul National University Hospital (IRB No. 1904-054-1027) and written consent forms were obtained from all parents or their legal representatives. All medical records were reviewed and home videos of patients with movement symptom were collected and analyzed independently by 2 pediatric neurologists.

### Whole exome sequencing and variant identification

Of the 6 families, 3 underwent trio-WES and the other 3 had only the probands sequenced. Genomic DNA was extracted from peripheral blood leucocytes using a QIAamp DNA Blood Midi Kit according to the manufacturer’s instructions (Qiagen, Valencia, CA, USA). WES procedures including exome capturing and sequencing were performed at Theragen Etex Bio Institute (Suwon, Korea). The sequenced reads were aligned to human reference genome patch 13 (GRCh37.p13) using a Burrows–Wheeler Aligner (version 0.7.15). Picard software (version 2.8.0), SAMtools (version 1.8), and a Genome Analysis Toolkit (GATK, version 4.1.4) were used for further data processing such as removal of polymerase chain reaction (PCR) duplicates, base recalibration, and variant quality control. All variants were called using the GATK HaplotypeCaller in GVCF mode, and the called variants were annotated using ANNOVAR and SnpEff. The pathogenicity of variants was evaluated according to the American College of Medical Genetics (ACMG) standards guidelines [14]. Segregation test was done for 3 proband-WES cases using Sanger sequencing.

### Amplicon sequencing

**Fig. 1:**
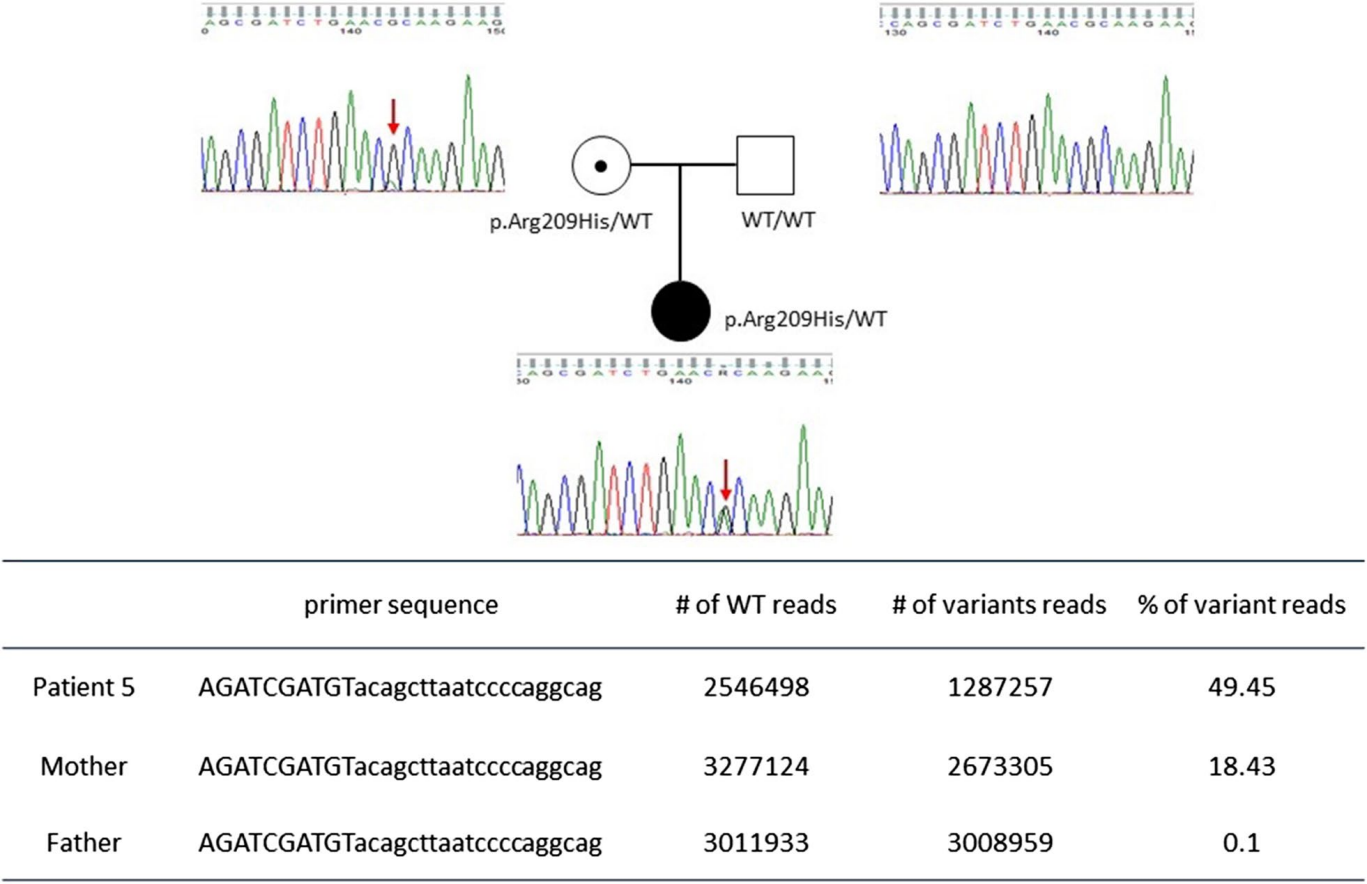
Pedigree and result of genetic testing of patient 5 who carried the variant p.Arg209His. Sanger sequencing analysis indicated the mother’s heterozygous peak. The result of sequential barcoded amplicon sequencing is described and the patient’s mother carried a somatic mutation

Amplicon sequencing was conducted for 1 family (patient 5) to identify parental mosaicism (Fig. 1). The primer was designed to amplify genomic region of interest. It can create a single amplicon of approximate 200 bp and the target position has a distance of 100 bp or less from the 5′ end. Six nucleotide barcode and adaptor sequences were added to the 5′ end of the primers to identify family members. PCR procedures were performed as previously described in detail [15]. Next-generation sequencing was performed using a dual indexing strategy and PCR free kit by Theragen Etex Bio Institute (Suwon, Korea).

## Results

### Overall clinical features

Three female and 3 male patients were enrolled and evaluated. All clinical features are summarized in Table 1. Five patients were referred to the clinic due to hypotonia or global developmental delay which was noticed at different ages (3 months old on median, range 0–75 months). Four patients (patients 2–5) had severely retarded development in motor, language, and social manner. Patient 4 showed weak crying and respiratory difficulty after birth, and was treated in a neonatal intensive care unit. She started unsteady gait without support at 4 years old, but no further achievement was shown till the most recent follow-up at her age of 8.8 years. Only 1 case (patient 4) had focal epilepsy, which started at 6 years old and was well controlled with valproate monotherapy. Patient 3 had neither movement disorder nor epilepsy. He presented as having infantile hypotonia and profound developmental delay. Generalized spasticity developed and progressed over time, dominantly on lower extremities.

**Table 1:** Clinical features of six patients with *GNAO1* variants

	Patient 1	Patient 2	Patient 3	Patient 4	Patient 5	Patient 6
Sex, age	Female, 16.9 y	Male, 7.2 y	Male, 3.3 y	Female, 8.8 y	Female, 7.7 y	Male, 4.0 y
*Genotype*						
Variant Inheritance Reference	p.Ala338del de novo novel	p.Glu246Lys de novo Saitsu et al. 2016	p.Ala301del de novo novel	p.Ala227Val De novo Saitsu 2016	p.Arg209His Maternal mosaicism Kulkani 2016	p.Arg206Leu de novo novel
Onset age	6.3y	3 m	3 m	Since birth	3 m	34 m
Initial symptom	Clumsiness on hands	Hypotonia	Hypotonia	Hypotonia	Hypotonia	Developmental delay
Motor development	Walk alone (18 m)	No achievement (near bed-ridden)	Sit up	Unsteady gait (4 y) No progression	Unsteady gait (5.8 y) Then regressed	Unsteady gait (2 y) No progression
Language development	Sentences Intellectual disability	No achievement	2 words	2 words	50 words, Then regressed	2 words
Epilepsy (onset) age)	No	No	No	Focal epilepsy (6Y)	No	No
EEG findings	Normal	Normal	Normal	Focal spikes	Normal	Normal
Movement disorder (onset age)	Myoclonus, focal dystonia (10Y)	Severe chorea Focal dystonia (2Y)	No	Hand stereotypi (NA)	Orofacial dyskinesia Chorea Focal dystonia (around 1Y)	Focal dystonia (2Y)
Others	Spasticity Scoliosis Difficulties on fine motor function	Progressive generalized spasticity	Progressive spasticity (lower extremity dominant)	−	Progressive spasticity	Ataxia
Brain MRI (performed age)	Normal (14 y)	Atrophy of bilateral head of caudate nucleus (6 y)	Normal (2.5 y)	Normal (6 Y)	Normal (5 y)	Normal (2.5 y)

y, years; m, months

### Phenotypic spectrum of movement disorder

Movement disorder was identified in 5 patients in a different manner. Patient 1 initially visited the rehabilitation clinic because of poor hand skills at 6 years old, but no further tests were given because her symptoms were quite subjective without any progression nor abnormalities on neurological examination. However, intermittent nonepileptic truncal myoclonus followed by focal dystonic gait was recognized at the age of 10 years. She underwent several genetic tests for dystonia including Segawa disease, but no sequence variants in known genes were noticed. Her symptoms had progressed slowly and spasticity of lower extremities and increased knee jerk was noted at the latest clinical follow-up (17 years old).

Patient 2 started his choreoathetosis around 2 years old. His chorea was accompanied by brief focal dystonia, which lasted all day long and disappeared during sleep. It was not much deteriorated with advancing age (see Additional file 1).

Patient 5 was initially reported to have intermittent hyperkinesia with brief jerking and truncal dystonia triggered by emotional upset which started at 1 year old (see Additional file 2). At the age of 5 years, orofacial dyskinesia developed and became prominent (see Additional file 3). Brief dystonia on her neck and shoulder also occurred and her movement presentations progressed over time (see Additional file 4, at 8 years old). Eventually she found it difficult to walk or crawl. Her movements showed acute exacerbations during febrile illness.

Patient 6 showed ataxia before 1 year of age without significant worsening. He started walking independently at 16 months old, but his gait has remained unstable till the most recent follow-up. Brief focal dystonia of lower extremities was also noted during walking (see Additional files 5 and 6, at 24 and 27 months old, respectively).

### Mutation analysis and genotype–phenotype association

Six different variants from 6 patients were identified using WES (Table 1). Patients 1–3 underwent trio-WES whereas patients 4–6 had WES for proband only. All variants were classified as pathogenic or likely pathogenic according to the ACMG guidelines. Five variants were confirmed to be de novo mutations, but patient 5 inherited her variant from her mother who carried the mosaic variant, a state identified through amplicon sequencing (Fig. 1). In this cohort, 4 variants (from patients 2, 4, 5, and 6) were located in a mutational hot spot and 3 of them (p.Glu246Lys of patient 2, p.Ala227Val of patient 4, p.Arg209His of patient 5) were reported previously [2, 4, 6, 7]. We reviewed previously reported cases and compared detailed phenotypes (Table 2). Patients with the variant of p.Glu246Lys or p.Arg209His showed quite homogeneous phenotypes: e.g., infantile hypotonia, profound development delay, or severe choreoathetosis started in early childhood. In particular, patients with p.Arg209His were reported to have severe exacerbation and required multiple admissions.

**Table 2:** Phenotype review of the patients with the variant p.Glu246Lys and p.Arg209His

No	Reference	Age/sex	Initial symptom (onset age)	Epilepsy (onset age)	Movement disorder (onset age)	Max motor achievement	Max speech achievement	Brain MRI
*p.Glu246Lys*								
1	This report (patient 2)	M/7.2 y	Hypotonia (3 m)	None	Chorea, dystonia (2 y)	None	None	Atrophy of bilateral head of caudate nucleus (6 y)
2	Saitsu^7^	F/13 y	Developmental delay (4 m)	None	Severe athetosis (NA)	None	None	Normal (12 y)
3*	Ananth^6^	M/5.5 y	Hypotonia (3 m)	None	Chorea (4 y)	None	None	Normal (12 m)
4*	Ananth^6^	F/5.5 y	Hypotonia (3 m)	None	Chorea (4 y)	None	None	Global atrophy (5.5 y)
5	Ananth^6^	F/10.3 y	Hypotonia (6 m)	None	Chorea (4 y)	None	None	Global atrophy, T2 hypointensity in globus pallidi (9 y)
6	Ananth^6^	M/15 y	Hypotonia (5 m)	None	Chorea (4 y)	None	None (simple non-verbal communication)	T2 hypointensity in globus pallidi (14 y)
7†	Schorling^4^	M/8 y	Myoclonic twitching (1 m)	None	Myoclonus (1 m), Dystonia (2 y)	None	NA	Normal (18 m)
8†	Schorling^4^	F/3 y	Developmental delay (5 m)	Focal epilepsy (7 m)	Dystonia (NA)	Head control	NA	Atrophy, thin corpus callosum (2 y)
*p.Arg209His*								
1	This report (patient 5)	F/7.7 y	Hypotonia (3 m)	None	Orofacial dyskinesia Chorea Focal dystonia Myoclonus (2 y)	Stand (regressed)	50 words	Normal (5 y)
2‡	Kulkani^2^	M/8 y	Hypotonia (18 m)	None	Sever Chorea Athetosis (34 m)	NA	NA	Normal (7 y)
3‡	Kulkani^2^	M/6 y	Hyperkinesia (2 y)	None	Severe Chorea Athetosis (2 y)	NA	NA	Normal (6 y)
4	Ananth^6^	M/16 y	Hypomotor (6 m)		Chorea (3 y)	Head control	Monosyllable words	Global atrophy (15 y)

^*^They were dizygotic twins from non-consanguineous parents

^†^They were siblings from non-consanguineous parents

^‡^They were siblings from on-consanguineous pare

## Discussion

Movement disorder is difficult to evaluate, especially in children, because they are in a developmental process and their symptoms evolve over time. Comprehensive phenotyping is quite important for diagnosis of these patients, even in the genomic era. In our cohort, all except 1 patient had multiple genetic testing including diagnostic exome sequencing and gene panel sequencing before participation in KUDP. After the diagnosis, we reviewed the patient’s clinical course including previous videos. Patients 1 and 3 showed atypical presentations. We also found some quite unique presentations of patients with some recurrent *GNAO1* variants (p.Glu246Lys and p.Arg209His). As previously reported, patients initially presented with profound infantile hypotonia and developmental delay, and severe choreoathetosis movement developed in all patients during early childhood [2, 4, 6, 7]. Patient 2 in our cohort showed movement symptoms since he was about 2 years old. The symptoms persisted, but were not much progressed or suddenly exacerbated, as reported in patients with a p.Glu246Lys variant. Patient 5 was reported to have orofacial dyskinesia starting at 4 years old, but was eventually found to have early hyperkinetic movement. She was also admitted several times for pneumonia and accompanying aggravation of neck dystonia as in the previous cases [2, 6]. This indicated a quite homogeneous and characteristic clinical course with high suspicion of GNAO1 encephalopathy. Most of those patients visited a clinic for their hypotonia or developmental delay, which are nonspecific and common for a pediatric neurologist. However, if we noticed unexplained early onset dystonia or chorea as well as severe developmental delay, *GNAO1* might be initially considered as a genetic cause. Early diagnosis based on clinical presentation is important because of additional treatment options such as deep brain stimulation and tetrabenazine for severe movement phenotypes [2, 9, 16].

It is obvious that a genotype–phenotype correlation exists, at least in certain loci. Many functional studies were performed, especially for recurrent mutations to date. Some variants work as GOM, whereas others work as loss-of-function mutations [1, 2, 11]. Different functional alteration may contribute to different phenotypes of GNAO1 encephalopathy [11, 12]. Further studies are expected to evaluate other variants in *GNAO1* and its related pathway, which will allow us to know more about the disease and its possible treatment.

We verified maternal mosaicism from 1 family (patient 5) by additional amplicon sequencing. We suspected mosaicism in this patient, based on confirmative Sanger sequencing for the patient’s mother, indicating a low heterozygous peak. This verification is crucial for the family counselling. Three familial cases were already reported in spite of a small number of total patients, which suggested parental mosaicism might be common in GNAO1 encephalopathy [2, 4, 6, 17]. Therefore, testing for the mosaicism should be conducted if parents have plan to have another child.

## Conclusions

This study reported 6 cases of GNAO1 encephalopathy focusing on their movement phenotypes with their video clips (Additional files). Early-onset chorea with profound developmental problems is quite characteristic for patients with a movement-dominant phenotype. We also reported atypical and novel findings for GNAO1 encephalopathy including proven mosaicism in parents, which is important to guide genetic counselling.

## Supplementary information

Additional file 1: Chreoathetosis with multifocal brief dystonia in Patient 2 (at 4 years old).

Additional file 2: Hyperkinesia and choreoathetosis in Patient 5 (at 1 year old).

Additional file 3: Orofacial dyskinesia in Patient 5 (at 4 years old).

Additional file 4: Sustained choreoathetosis with spasticity on lower extremities and orofacial dyskinesia (at 8 years old).

Additional file 5: Focal mild dystonic gait (at 24 months old).

Additional file 6: Focal dystonic gait with mild ataxia (at 27 months old).

## Data Availability

All data generated or analyzed for the study are available from the corresponding author upon reasonable request.
